# Highly stable mesoporous silica nanospheres embedded with FeCo/graphitic shell nanocrystals as magnetically recyclable multifunctional adsorbents for wastewater treatment†

**DOI:** 10.1039/c7ra12240c

**Published:** 2018-01-03

**Authors:** Yonghoon Hong, Da Jeong Kim, In Ae Choi, Mou Pal, Gaehang Lee, Ki Min Nam, Won Seok Seo

**Affiliations:** Department of Chemistry, Sogang University Seoul 04107 Republic of Korea wsseo@sogang.ac.kr; Instituto de Física, BUAP, Ciudad Universitaria Av. San Claudio y Blvd. 18 Sur Col. San Manuel C.P. 72570 Puebla Mexico; Korea Basic Science Institute, University of Science and Technology Daejeon 34133 Republic of Korea ghlee@kbsi.re.kr; Department of Chemistry, Mokpo National University Jeonnam 58554 Republic of Korea namkimin.chem@gmail.com

## Abstract

Highly stable and magnetically separable mesoporous silica nanospheres (MSNs) embedded with 4.6 ± 0.8 nm FeCo/graphitic carbon shell nanocrystals (FeCo/GC NCs@MSNs) were synthesized by thermal decomposition of metal precursors in MSNs and subsequent methane CVD. The FeCo/GC NCs@MSNs had a high specific surface area (442 m^2^ g^−1^), large pore volume (0.65 cm^3^ g^−1^), and tunable size (65 nm, 130 nm, and 270 nm). Despite the low magnetic metal content (8.35 wt%), the FeCo/GC NCs@MSNs had a sufficiently high saturation magnetization (17.1 emu g^−1^). This is due to the superior magnetic properties of the FeCo/GC NCs, which also enable fast magnetic separation of the nanospheres. The graphitic carbon shell on the FeCo NCs not only protects the alloy core against oxidation and acid etching in 35% HCl_(aq)_, but also facilitates non-covalent, hydrophobic interactions with the hydrocarbon chains of organic dyes such as methyl orange and methylene blue. Surface functionalization of the FeCo/GC NCs@MSNs with thiol groups provides efficient capacity for binding with Hg^2+^ ions. We have shown that the thiol-functionalized FeCo/GC NCs@MSNs (FeCo/GC NCs@MSNs-SH) work as multifunctional adsorbents for organic dyes (target organic pollutants) and Hg^2+^ ions (target inorganic pollutant). We also demonstrated that the FeCo/GC NCs@MSNs-SH are excellent recyclable adsorbents for methyl orange.

## Abstract 2

We report the first synthesis of highly stable and efficiently recyclable multifunctional adsorbents containing FeCo/GC nanoparticles with the strongest magnetic properties.

## Graphical abstract

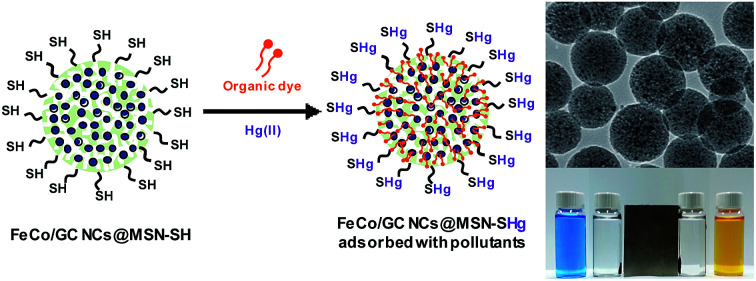

## 1. Introduction

Efficient removal of inorganic and organic toxic pollutants including heavy metal ions, dyes, and hazardous organic compounds from wastewater has been of great interest in the past decade.^1–6^ Many techniques have been used to remove harmful substances, including chemical precipitation, ion-exchange, adsorption, and reverse osmosis.^7–9^ Among them, adsorption is the most extensively used technique due to its simplicity and low cost. In the search for inexpensive and efficient adsorbents for wastewater cleanup, a large number of inorganic materials including activated carbon, graphene, silica, zeolites, and clays have been investigated.^10–12^ However, in some cases, low loading capacity, poor selectivity for heavy metal ions, and weak binding affinities have limited their widespread application in the purification of wastewater.^13,14^ In this context, mesoporous silica materials meet the requirements for highly efficient adsorbents because they possess large surface areas and pore volumes, good chemical stability, high dispersibility, and interconnected frameworks with active sites useful for functionalization or other chemical modifications.^15–18^ Modification of the surface chemical properties of mesoporous silica can improve its capacity for binding with heavy metal ions^19–22^ or organic dyes^23,24^ from contaminated water. Carbon-based nanomaterials with high surface area, such as graphene, carbon nanotubes, and porous carbon have also been widely used as adsorbents for the removal of organic pollutants because of the hydrophobic properties of their surfaces.^25,26^ However, the hydrophobic nature of carbon is disadvantageous with respect to its interaction with aqueous media.^27^

Adsorbents possessing magnetic properties are advantageous because they provide for convenient separation of the adsorbents from media after adsorption through simple magnetic attraction.^28–31^ Magnetic silica or carbon nanocomposites with various structures have been investigated as adsorbents for heavy metal ions or organic dyes.^32^ The magnetic materials in adsorbents have mostly been iron-containing metal oxides.^33^ However, these are not desirable in some cases due to their low magnetization (*M*_s_ of bulk magnetite ≤ 92 emu g^−1^)^34^ or inherent instability in acidic media.^33^ It might be impossible to remove magnetic adsorbents effectively from solution unless they contain large amounts of magnetic metal oxides. Furthermore, magnetic metal oxides might dissolve during adsorption in wastewater containing highly reactive species such as strong acids. This would cause the loss of attraction to external magnetic fields and, more significantly, further contaminate the water by releasing metal ions.^26,35^ To circumvent these limitations, zero-valent metals such as Fe, Co, and FeCo with strongest magnetic properties (*M*_s_ of a bulk FeCo = 235 emu g^−1^)^36^ were applied as magnetic materials in adsorbents, mainly with some form of carbon-containing material. An Fe nanoparticle–graphene composite prepared *via* reduction of graphene oxide and iron chloride showed great potential as an efficient adsorbent of lead.^37^ Mesoporous Fe_7_Co_3_/carbon nanocomposite prepared *via* a co-casting method exhibited excellent performance for the adsorption of bulk dyes.^38,39^ However, these synthesis methods still cannot produce magnetic-metal nanoparticles that are perfectly resistant against acid etching and oxidation. Carbon-coated Co nanoparticles prepared by ethanol CVD were used as adsorbent for organic compounds.^40^ However, the magnetic nanoparticles thereby obtained were not very uniform in particle size and were coated with several carbon shells, which made them unsuitable for adsorption applications. Furthermore, magnetic-metal nanoparticles have yet to be applied for use as multifunctional adsorbents for both organic and inorganic pollutants owing to their carbon-based structures. Therefore, development of highly stable and efficiently recyclable multifunctional adsorbents with magnetic-metal nanoparticles prepared by a facile synthetic approach is truly needed.

**Fig. 1:**
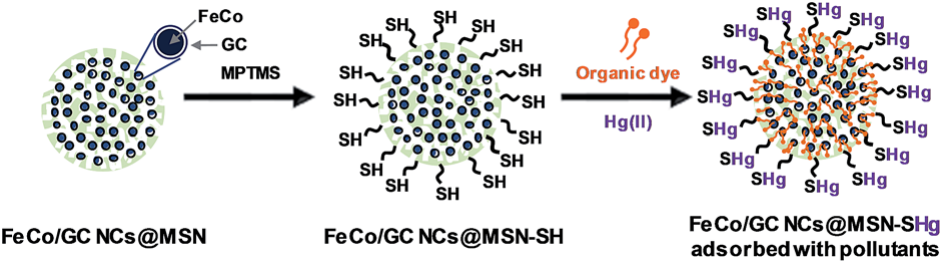
Schematic illustration for the preparation of FeCo/GC NCs@MSNs-SH and the multifunctional adsorption of organic dyes and mercury(ii) ions.

Herein, we describe mesoporous silica nanospheres (MSNs) incorporated with FeCo/graphitic carbon shell nanocrystals (FeCo/GC NCs) grown inside the pores of the silica nanospheres (FeCo/GC NCs@MSNs). As illustrated in Fig. 1, the reaction of the hydroxyl groups on the FeCo/GC NCs@MSNs with (3-mercaptopropyl)trimethoxysilane (MPTMS) gives thiol-functionalized FeCo/GC NCs@MSNs (FeCo/GC NCs@MSNs-SH) that have sites efficient for binding Hg^2+^ ions. The superior magnetic properties of the FeCo/GC NCs were exploited for simple, fast, efficient separation of the nanospheres from the matrix solutions using a magnet. We have tested the functionality of the FeCo/GC NCs@MSNs-SH as potential adsorbents in water pollution control experiments by selecting methyl orange (MO) and methylene blue (MB) as target organic pollutants, and Hg^2+^ ions as a target inorganic pollutant. Fig. 1 illustrates the adsorption mechanisms for the target pollutants using our FeCo/GC NCs@MSNs-SH. This is the first demonstration of such highly stable and efficiently recyclable multifunctional adsorbents containing metal nanoparticles with the strongest magnetic properties.

## 2. Experimental

### 2.1. Materials

Cetyltrimethylammonium bromide (CTAB, 98+%, Aldrich), triethanolamine (TEA, 99%, Aldrich), tetraethyl orthosilicate (TEOS, 98%, Aldrich), Fe(NO_3_)_3_·9H_2_O (99.99%, Aldrich), Co(NO_3_)_2_·6H_2_O (99.999%, Aldrich), hydrochloric acid (35%, Kanto), MPTMS (95%, Aldrich), Hg(NO_3_)_2_·H_2_O (98+%, Aldrich), methyl orange (MO, Fluka), and methylene blue (MB, Aldrich) were used without further purification. All other reagents purchased from commercial sources were used as obtained without further purification.

### 2.2. Synthesis of mesoporous silica nanospheres (MSNs)

Discrete and fully mesoporous silica nanospheres were prepared through a modified Stöber process.^41^ CTAB (0.400 g, 1.10 mmol) and TEA (6.00 mL, 45.2 mmol) were added to 70 mL solution (ethanol and water), and then dissolved completely under magnetic stirring at room temperature. 15/55, 17/53, and 20/50 volume ratios of ethanol/water were applied to prepare MSNs with average diameters of 65 nm, 130 nm, and 270 nm, respectively. To this solution, TEOS (2.80 mL, 12.5 mmol) was added and continuously stirred for 2 h at 80 °C. The resulting product was collected by centrifugation and washed repeatedly with distilled water and ethanol, followed by drying in air. For CTAB extraction, the synthesized solid product was subjected to refluxing in 100 mL of ethanol solution containing 2 mL of 35% HCl_(aq)_ for 3 h. The resulting MSNs were collected by centrifugation, repeatedly washed with distilled water and ethanol.

### 2.3. Synthesis of FeCo/GC NCs@MSNs

130 nm MSNs (0.500 g) was sonicated in methanol (50 mL) for 10 min. Then, Fe(NO_3_)_3_·9H_2_O (0.207 g, 0.513 mmol) and Co(NO_3_)_2_·6H_2_O (0.113 g, 0.387 mmol) were added to the mixture and subjected to sonication for another 1 h. After removal of methanol through evaporation and drying at 80 °C, we ground the powder and typically used 0.500 g for methane CVD in a tube furnace. The powder precursors were heated in a H_2_ flow to reach 900 °C and then subjected to a methane flow of 400 cm^3^ min^−1^ for 5 min. After cooling, the sample was etched in a 10% HCl_(aq)_ solution to remove any metal impurities including incompletely capsulated metal nanoparticles. The resulting FeCo/GC NCs@MSNs were collected by centrifugation and thoroughly washed with distilled water and ethanol, respectively.

### 2.4. Synthesis of FeCo/GC NCs@MSNs-SH

FeCo/GC NCs@MSNs (0.100 g) was dispersed in dry toluene (10 mL). Then, MPTMS (2.00 mL) was added into the mixture and refluxed under a nitrogen atmosphere for 24 h. Thiol-functionalized FeCo/GC NCs@MSNs (FeCo/GC NCs@MSNs-SH) were collected by centrifugation, and washed with dry toluene.

### 2.5. Characterization

Transmission electron microscopy (TEM) with selected area electron diffraction (SAED) patterns and energy dispersive analyses of X-ray emission (EDX) were performed on a JEOL JEM-2100F, operated at an acceleration voltage of 200 kV. X-ray diffraction (XRD, Rigaku Mini flexII) patterns of the nanocomposites were obtained using Cu-Kα radiation at 30 kV and 15 mA. Nitrogen sorption isotherms were recorded using a BELSORP-max instrument with nitrogen. The BET (Brunauer–Emmett–Teller) specific surface areas were calculated using adsorption data in a relative pressure range of *p*/*p*_0_ = 0.05–0.30. Pore size distribution curve was calculated from the adsorption and desorption branches of the corresponding N_2_ isotherm by using BJH (Barrett–Joyner–Halenda) method. Prior to each measurement the sample was degassed at 300 °C for 24 h under vacuum to remove all the impurities completely. The concentrations of dye remaining in the solution were determined using a ultraviolet-visible-near-infrared spectrometer (JASCO V-660). Fourier transform infrared (FT-IR) spectra of the samples in KBr were collected with a FT-IR spectrometer (THERMO ELECTRON Co., AVATAR 330). Thermogravimetric analysis (TGA, TA INSTRUMENT 2050) was carried out in the range of 30–800 °C under nitrogen flow at a heating rate of 10 °C min^−1^. Elemental analyses were performed with a Perkin-Elmer 2400 Series II analyzer. The concentrations of heavy metal ions in the solutions were measured by using an ICP-AES (Inductively Coupled Plasma-Atomic Emission Spectrometer) in ANAPEX. The magnetic measurements were performed on a superconducting quantum interference device-vibrating sample magnetometer (SQUID-VSM, Quantum Design MPMS). DC susceptibility and hysteresis measurements were recorded for powdered samples in a gelatin capsule. The temperature was varied between 2 and 300 K according to a zero field cooling/field cooling (ZFC/FC) procedure at 100 Oe, and the hysteretic loops were obtained in a magnetic field varying from +7 to −7 T.

### 2.6. Adsorption of organic dyes

In a typical dye-adsorption experiment, an aqueous dye stock solution (100 ppm) was prepared by dissolving a dye in deionized water. Aqueous dye solutions with different concentrations (5–60 ppm) were then prepared by successive dilution of the stock solution with deionized water. Adsorbents (20 mg) were put into each dye solution (20 mL). The mixture was shaken at room temperature until the equilibrium was reached (typically 3 h). After that, the powder adsorbent was separated from the suspension using a NbFeB magnet and the dye concentration in the clear supernatant was determined using a UV-vis spectrophotometer at the maximum absorbance peaks of MB (*λ* = 664 nm) and MO (*λ* = 464 nm). The equilibrium adsorption capacities (*Q*_e_) were determined according to the following formula*Q*_e_ = (*C*_i_ − *C*_e_)*V*/*m*wherein *C*_i_ is the initial concentration, *C*_e_ is the equilibrium concentration, *V* is the volume of the liquid phase, *m* is the mass of the adsorbent. To study the effect of contacting time on adsorption, we continuously shook the mixture of an adsorbent (20 mg) and 50 ppm MB or 25 ppm MO solution (20 mL) and took an aliquot of 1 mL at different times, and then quickly separated the adsorbent from the aliquot by using a magnet (typically within 30 s). The pH values of the solutions were adjusted from pH 2 to 11 by adding 0.1 M HCl and 0.1 M NaOH solutions.

### 2.7. Desorption of MO

Desorption of MO was carried out by immersing the dye-adsorbed FeCo/GC NCs@MSNs-SH into methanol (50 mL) containing 0.28% aqueous ammonia. After soaking for 30 min, the dye-desorbed FeCo/GC NCs@MSNs-SH were collected using a magnet, resulting in almost clean regenerated FeCo/GC NCs@MSNs-SH which were then used in the next adsorption experiment.

### 2.8. Decoloration

FeCo/GC NCs@MSNs-SH (20 mg) was added into dye solution (20 mL). The concentration of each dye solution was 10 ppm for MB and 5 ppm for MO. After 5 min, the dye-adsorbed FeCo/GC NCs@MSNs-SH were collected using a magnet (typically within 1 min), resulting in almost clear and colorless solutions.

### 2.9. Adsorption of mercury(ii) ions

A solution of 1000 ppm Hg^2+^ ion was prepared by dissolving mercury(ii) nitrate in an aqueous solution of the desired pH. The pH values of the solutions were adjusted using a 0.1 M HCl solution. Each adsorbent (50 mg) was shaken in the Hg^2+^ solution (50 mL) at room temperature for 12 h, respectively. After separation of the adsorbents, the amounts of residual Hg^2+^ ions in the supernatant were measured using an ICP-AES.

## 3. Results and discussion

We synthesized FeCo/GC NCs@MSNs following the method established for synthesis of FeCo/GC NCs in MSNs, with slight modification.^42,43^ In our previous report, we used MSNs that were not discrete and size-controlled.^42^ Herein, we synthesized discrete and fully mesoporous silica nanospheres with controllable size, and used them not only as support for the synthesis of FeCo/GC NCs but also as anchors for the binding of mercury(ii) ions through thiol groups grafted onto the silica surface. The discrete MSNs were synthesized using a surfactant templating approach with TEA as a basic catalyst instead of more commonly used sodium hydroxide.^41^ TEA is supposed to serve as a complexing agent for silicate species and additionally as an encapsulator for mesostructured nanospheres, limiting the growth and aggregation of particles.^44^ On the other hand, the cationic surfactant CTAB acts as a surfactant for silicate species as well as a structure directing agent for the formation of mesopores.^45^ Under alkaline condition, the self-assembly of CTAB and anionic silicate species takes place to produce mesoporous silica. The average size of the MSNs was tunable by changing the ratio of the solvents (ethanol/water). As the ethanol/water ratio decreases, smaller MSNs were synthesized. The tendency to obtain smaller MSNs with increasing water content can be explained by acceleration of the hydrolysis of TEOS and the water-forming condensation reaction in the presence of a larger amount of water. This produces a larger number of silica seeds and eventually, smaller silica nanospheres.^46^ The MSNs were further loaded with metal salts (Fe(NO_3_)_3_·9H_2_O, Co(NO_3_)_3_·6H_2_O) by impregnation in methanol, followed by evaporation of the solvent. The metal precursors were preferentially incorporated into the mesoporous channels of MSNs. During the heating process under H_2_ flow at 900 °C, bimetallic FeCo NCs were formed. Further methane CVD at that temperature promoted deposition of a graphitic carbon layer over the FeCo NCs grown inside the mesopores.

**Fig. 2:**
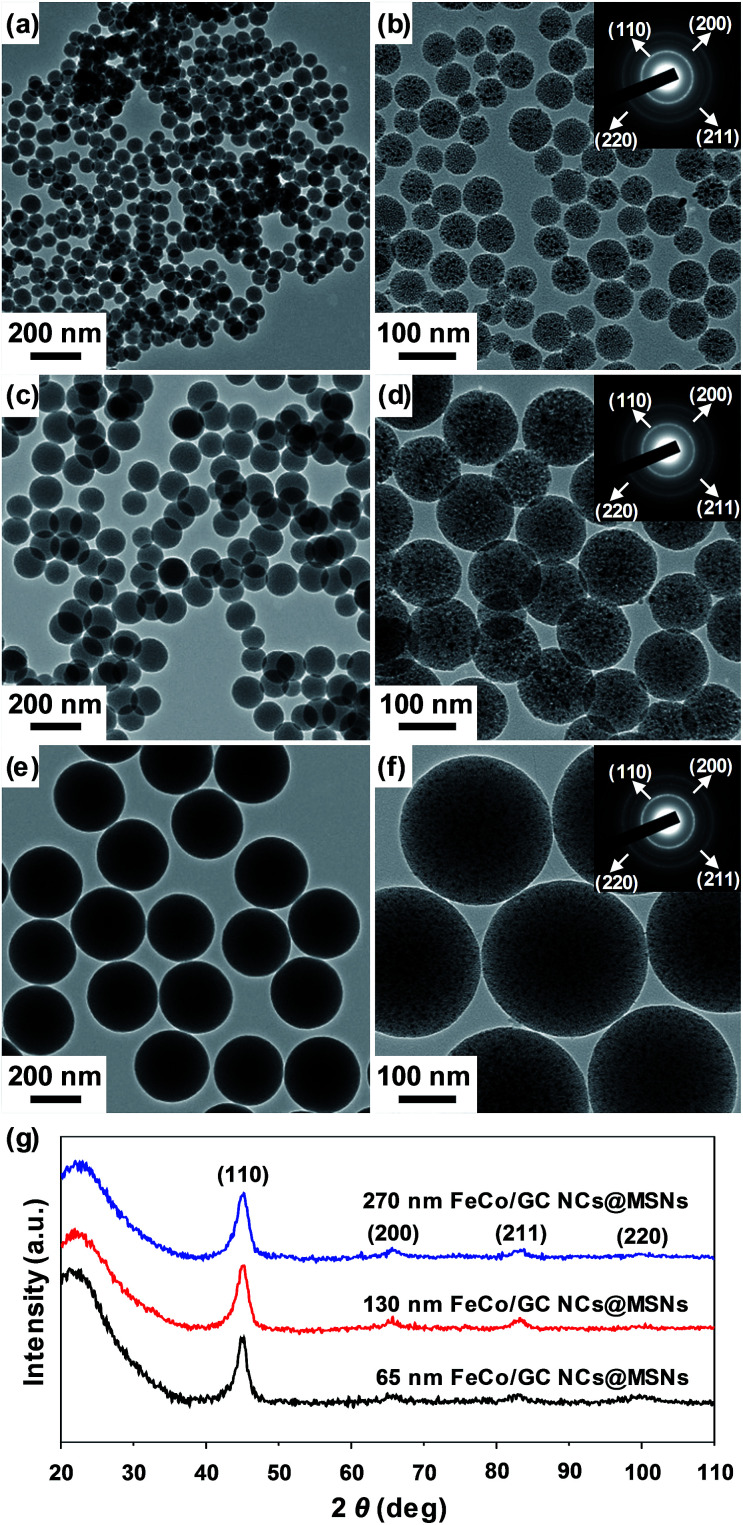
TEM images of (a, b) 65 nm, (c, d) 130 nm, and (e, f) 270 nm MSNs (a, c, e), and FeCo/GC NCs@MSNs (b, d, f). Insets in Fig. 2b, d, and f are SAED patterns. (g) XRD patterns of 65 nm, 130 nm, and 270 nm FeCo/GC NCs@MSNs.

Fig. 2a, c, and e show TEM images of MSNs of different sizes. When 15/55, 17/53, and 20/50 volume ratios of ethanol/water were used, MSNs with average diameters of 65 ± 18 nm (Fig. 2a), 130 ± 17 nm (Fig. 2c), and 270 ± 15 nm (Fig. 2e) were formed, respectively. Fig. 2b, d, and f show TEM images of the corresponding FeCo/GC NCs@MSNs for the MSNs of different sizes. The tiny black spots, which correspond to FeCo/GC NCs, were found to be well dispersed in the MSNs. When 0.9 mmol of metal precursors was loaded on 0.5 g of MSNs, FeCo/GC NCs with about the same average diameters (4.6 ± 0.8 nm) were formed for the three different MSN samples. This means that the metal precursors were homogeneously internalized within the silica channels regardless of the MSN size. The mean size and standard deviation of the FeCo/GC NCs were measured by TEM for ∼500 NCs obtained after treatment of the FeCo/GC NCs@MSNs with HF to dissolve the MSNs (Fig. S1, ESI†). XRD (Fig. 2g and S2 (ESI†)) and electron diffraction (insets of Fig. 2b, d, and f) techniques were used to identify the crystal structure of the FeCo core for the FeCo/GC NCs. The well-resolved XRD peaks and electron diffraction patterns well matched (110), (200), (211) and (220) reflections of a body-centered-cubic (bcc) phase of FeCo alloy. The crystallite sizes determined for the (110) peaks of the XRD data using the Debye–Scherrer equation^47^ were all the same (4.5 nm), which is in good agreement with the mean diameters determined from the TEM images.

We employed a 57 : 43 molar ratio of Fe and Co metal precursors to synthesize FeCo/GC NCs with a ratio of about 1 : 1 of Fe and Co metal content.^42,43^ EDX analysis for the emission of the samples shows Fe/Co ratios of 53 : 47, 52 : 48, and 52 : 48 for the 65 nm, 130 nm, and 270 nm FeCo/GC NCs@MSNs, respectively (Fig. S3, ESI†). The resulting 4.6 ± 0.8 nm FeCo/GC NCs with slight Fe richness have been reported to possess a single bcc FeCo phase and significantly high saturation magnetization values (>200 emu g^−1^ at room temperature), which makes them suitable for magnetic separation.^42,43,48^ The magnetic characterization of the FeCo/GC NCs@MSNs was investigated using a SQUID-VSM. As seen in Fig. S4a (ESI†), the temperature-dependent magnetization curves under ZFC and FC conditions show that the blocking temperature (*T*_B_) of the 130 nm FeCo/GC NCs@MSNs is 249 K at 100 Oe. Also, the field-dependent magnetization curve of the FeCo/GC NCs@MSNs measured at 300 K shows no hysteresis loop (Fig. S4b, ESI†), indicating the characteristic of a superparamagnetic system. Moreover, despite the low magnetic metal content (8.35 wt%) of the FeCo/GC NCs@MSNs, they have a very high saturation magnetization of 17.1 emu g^−1^, which is equal to the value of the samples with 20 wt% iron oxides. Note that FeCo/GC NCs not only have ∼3 times higher saturation magnetization values than those of iron oxide NCs, but also exhibit superior chemical stability against acid etching in contrast to magnetic metals without graphitic carbon shells, or metal oxides such as iron oxides and ferrites.^36^ In fact, the 130 nm FeCo/GC NCs@MSNs were very stable in a 35% HCl solution over a one-week monitoring period and was very quickly attracted when the sample was placed next to a cubic magnet. In contrast, 130 nm FeCo NCs@MSNs, in which the FeCo NCs were not encapsulated with carbon shells, turned the solution green right after addition of the HCl solution owing to the etching of Fe and Co (Fig. S5a and b, ESI†). The FeCo/GC NCs@MSNs also showed high stability in a strongly basic NaOH solution (pH 11) over a one-week monitoring period (Fig. S5c, ESI†). The TEM images of the FeCo/GC NCs@MSNs aged in the harsh acid and base solutions for a week (Fig. S5d and e, ESI†) proved that the samples remained the same after the aging, supporting the claim to stability.

**Fig. 3:**
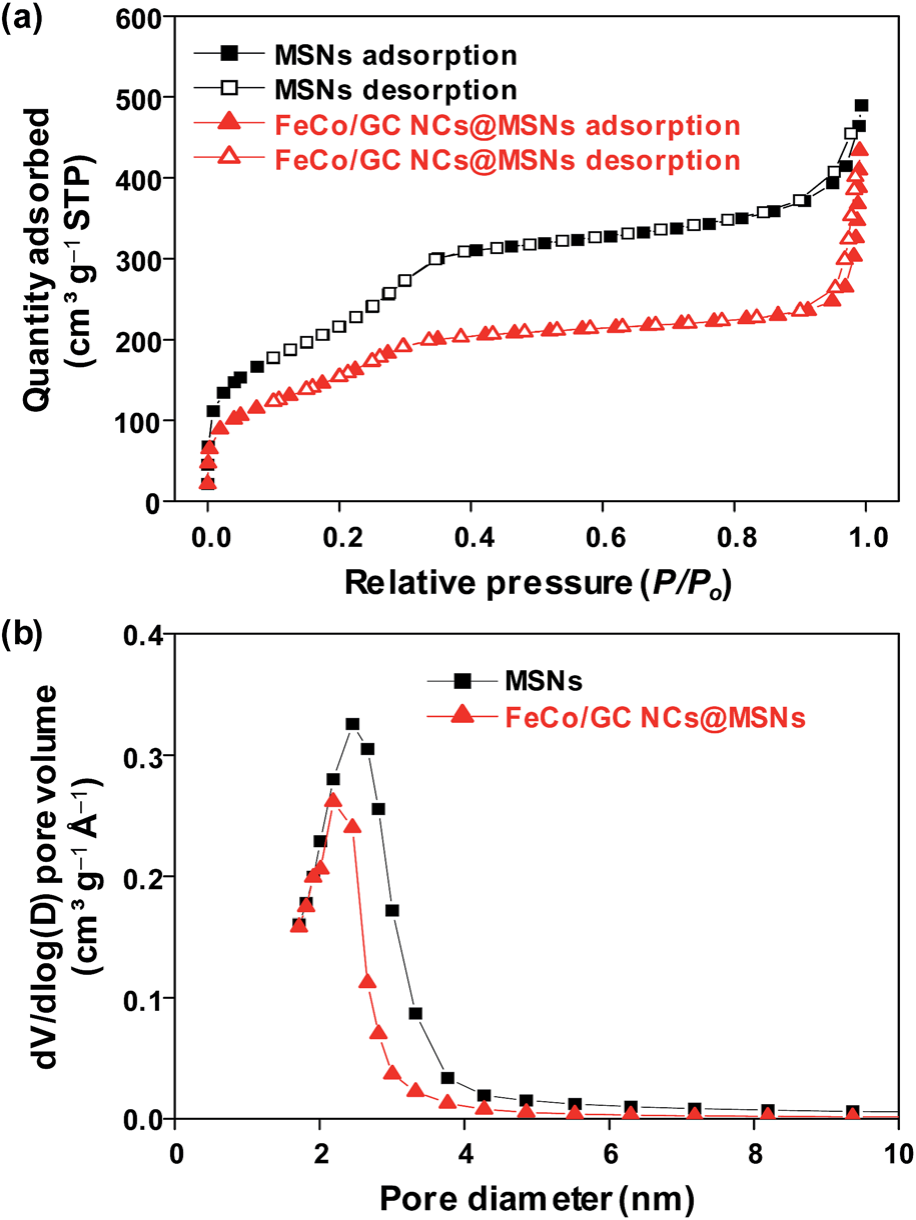
(a) Nitrogen sorption isotherms and (b) pore size distributions of 130 nm MSNs and FeCo/GC NCs@MSNs.

The surface area and the pore size distributions of the 130 nm MSNs and FeCo/GC NCs@MSNs were studied using N_2_ adsorption–desorption isotherms (Fig. 3). Table S1 (ESI†) summarizes the physisorption data. Both samples show a type IV adsorption isotherm with a H1-type hysteresis loop, which is characteristic of mesoporous silica.^49^ The BET surface area, total pore volume, and calculated average pore diameter for the MSNs were 661 m^2^ g^−1^, 0.73 cm^3^ g^−1^, and 2.45 nm, respectively. These were found to decrease to 442 m^2^ g^−1^, 0.65 cm^3^ g^−1^, and 2.19 nm after the loading of FeCo/GC NCs into the mesopores. This suggests that the nano-sized pores of the silica were partially blocked by the FeCo/GC NCs. The average size of the FeCo/GC NCs exceeded the pore size, which means that the pore walls must be deformed at the locations of NCs because the NCs grow larger than the pore size. This deformation prevented complete occlusion of the pores. Therefore, we expect that our FeCo/GC NCs@MSNs possess surface area and porosity large enough for their potential use as adsorbents.

**Fig. 4:**
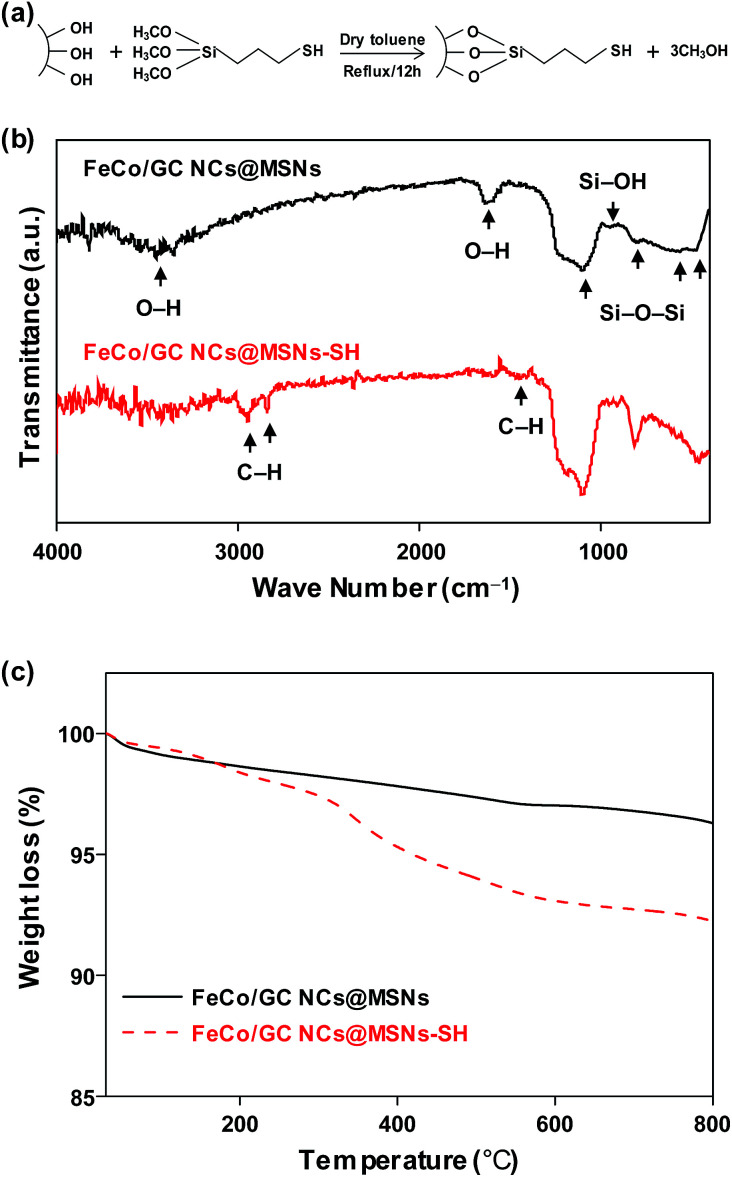
(a) Illustration of thiol functionalization reaction between MPTMS and silanol groups on the silica surface. (b) FT-IR and (c) TGA data of 130 nm FeCo/GC NCs@MSNs and FeCo/GC NCs@MSNs-SH.

Functionalization of the FeCo/GC NCs@MSNs was achieved by exploiting the large number of hydroxyl groups on the silica surface which largely facilitate the attachment of thiol groups; thereby providing binding sites for metal ions. Surface-immobilized thiol groups exhibit remarkable affinity for binding Hg^2+^ ions.^50,51^ A schematic representation of the reaction between MPTMS and silanol groups on the silica surface is shown in Fig. 4a. The thiol functionalized FeCo/GC NCs@MSNs (FeCo/GC NCs@MSNs-SH) was characterized by IR, elemental analysis, and TGA to confirm the attachment of thiol groups on the pore surface and to estimate the S content. As shown in Fig. 4b and S6 (ESI†), C–H stretching (bands at 2920 and 2840 cm^−1^) and bending (1470 cm^−1^) vibrations, that confirm the presence of methylene chain of mercaptopropyl groups, were observed only in the FT-IR spectrum of FeCo/GC NCs@MSNs-SH. The elemental analysis of FeCo/GC NCs@MSNs-SH (Table 1) gave a C/H/S ratio of 10.1 : 1.3 : 2.8, which roughly corresponds to the S content of 0.88 mmol per gram of sample. TGA further confirms the functionalization of MSNs with mercaptopropyl groups (Fig. 4c). The weight loss of FeCo/GC NCs@MSNs-SH at 800 °C was found to be 7.8%, which is 4.0% more than that of FeCo/GC NCs@MSNs. The increased loss is due to the decomposition of mercaptopropyl groups attached to the silica surface. The TEM images of 130 nm MSNs-SH and FeCo/GC NCs@MSNs-SH (Fig. S7a and b, ESI†) did not show any obvious difference from the original samples, in shape and size. This suggests that the thiol-functionalization process should not affect the particle morphology.

**Table 1:** Elemental analysis and Hg(ii) adsorption data for the adsorbents

Sample	Elemental analysis (wt%)				S content (mmol g^−1^)	Hg(ii) adsorbed (mg g^−1^)
	C	H	O	S		
MSNs	—	—	—	—	—	2.3
FeCo/GC NCs@MSNs	7.7	1.3	—	—	—	11.0
FeCo/GC NCs@MSNs-SHs	10.1	1.3	—	2.8	0.88	221.4

**Fig. 5:**
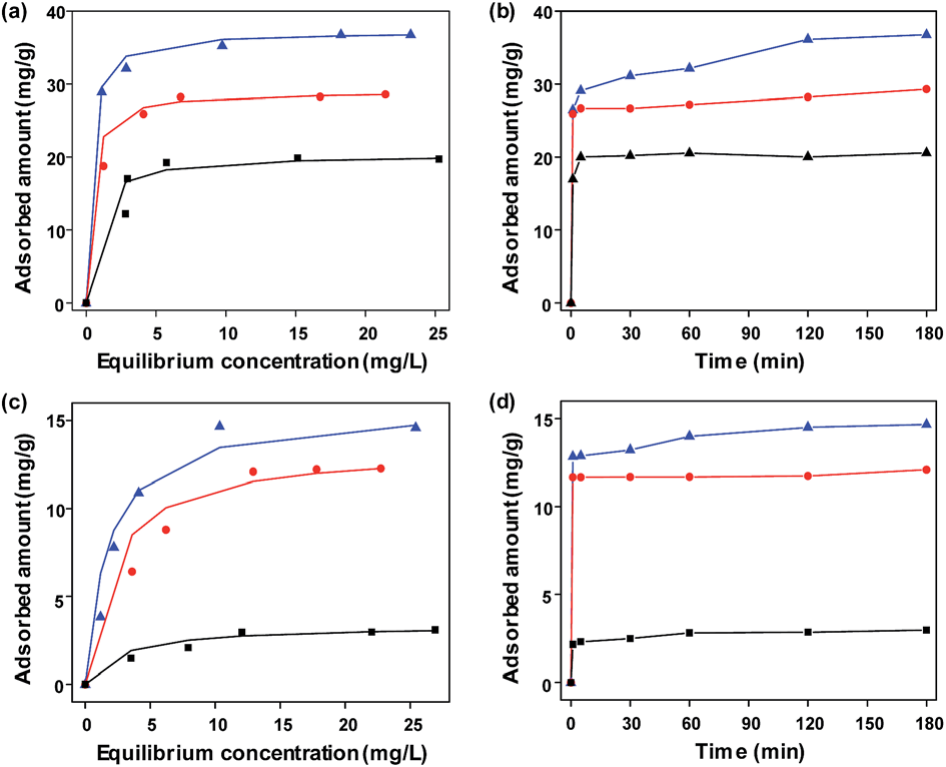
Adsorption isotherms of (a) MB and (c) MO for MSNs (squares), FeCo/GC NCs@MSNs (circles), and FeCo/GC NCs@MSNs-SH (triangles) in water (the solid lines represent the best fit of the experimental data to the Langmuir model). Adsorption curves of (b) MB and (d) MO for MSNs (squares), FeCo/GC NCs@MSNs (circles), and FeCo/GC NCs@MSNs-SH (triangles) in water as a function of contact time.

To investigate the functionality of the FeCo/GC NCs@MSNs-SH as an adsorbent for organic pollutants, we selected an acidic azo dye: MO, and a basic dye: MB as model pollutants and tested the adsorption properties. We also performed similar adsorption experiments using MSNs and FeCo/GC NCs@MSNs for comparison. Fig. 5a and c show the equilibrium adsorption isotherms of MO and MB on various adsorbents, respectively. The isotherms for FeCo/GC NCs@MSNs and FeCo/GC NCs@MSNs-SH belong to type I Langmuir isotherms; the amounts of adsorbed dyes dramatically increased at lower equilibrium concentration. This suggested a very high affinity between the dye molecules and the carbon shells of the FeCo/GC NCs embedded in the MSNs. The amounts adsorbed reached plateaus at a higher equilibrium concentration, reflecting saturated adsorption.^52^ The Langmuir adsorption equation (eqn (1)) was used to determine the mechanistic parameters associated with organic dye adsorption.

1 *C*_e_/*q*_e_ = 1/(*q*_max_*b*) + *C*_e_/*q*_max_

where *C*_e_ is the equilibrium concentration of the adsorbate (mg L^−1^), *q*_e_ is the equilibrium adsorption capacity of the adsorbate on the adsorbent (mg g^−1^), *q*_max_ is the maximum adsorption capacity (mg g^−1^), and *b* is the Langmuir adsorption constant (L mg^−1^), related to the free energy of adsorption. Table S2 and S3 (ESI†) summarize the Langmuir constants and the calculated correlation coefficients for MB and MO adsorption on various adsorbents. The Langmuir isotherms of FeCo/GC NCs@MSNs-SH fit quite well the experimental data (correlation coefficient *R*^2^ = 0.99) for both MB and MO adsorption. As shown in Table 2, the adsorption capacities of the FeCo/GC NCs@MSNs (28.6 mg g^−1^ for MB and 12.3 mg g^−1^ for MO) and FeCo/GC NCs@MSNs-SH (36.8 mg g^−1^ for MB and 14.6 mg g^−1^ for MO) were significantly higher than those of the MSNs (19.7 mg g^−1^ for MB and 3.1 mg g^−1^ for MO). This suggests that the adsorption of organic dyes should take place mainly *via* van der Waals or hydrophobic interactions between the graphitic shells and the hydrocarbon chains of the dye molecules, especially for MO. The high adsorption capacity of silica regarding MB can be attributed to the electrostatic interaction between negatively charged silica and positively charged MB in water.^53,54^ The FeCo/GC NCs@MSNs-SH showed slightly greater capacity than did the FeCo/GC NCs@MSNs. This can be explained by the new hydrophobic interaction occurring between hydrocarbon chains of the mercaptopropyl group and those of the dye molecules.^19,20^ It seems that the new interaction overrides the expected decrease in the interaction between the graphitic carbon and dyes due to the partial blocking of the pores caused by the functionalization of the silica surface with mercaptopropyl groups.

**Table 2:** Adsorption capacities of the adsorbents for dye molecules

*Q*_e_ (mg g^−1^)	Dye	
	Methylene blue	Methyl orange
MSNs	19.7	3.1
FeCo/GC NCs@MSNs	28.6	12.3
FeCo/GC NCs@MSNs-SH	36.8	14.6

In comparison with other carbon-based magnetic adsorbents in literatures with adsorption capacities ranging from 9.7–275.9 mg g^−1^ for MB and 5.0–102 mg g^−1^ for MO (Table S4, ESI†), our adsorbents display relatively low adsorption capacities. If we consider the graphitic carbon the only anchor for adsorption of dyes, and subtract the adsorption capacity of MSNs from that of the FeCo/GC NCs@MSNs, the adsorption capacity of the FeCo/GC NCs@MSNs would rise to 119 mg g^−1^ carbon for MO, which is among the highest adsorption capacities of graphitic carbon that has ever been reported.^55–58^ We expect that the adsorption capacities of the samples could be improved simply by increasing the content of graphitic carbon in the samples.

We further studied the effect of contact time on adsorption. Fig. 5b and d show plots of adsorbed amounts of dyes *versus* contact times with the adsorbents. The plots show sharp increases of the adsorbed amounts in the initial period, indicating rapid uptake of dye at the beginning, then slowing down until finally reaching equilibrium. We additionally studied the effect of solution pH (pH = 2, 4, 7, 9, and 11) on adsorption capacity. The maximum adsorptions of MB and MO onto the FeCo/GC NCs@MSNs-SH were observed at a neutral solution (pH = 7) and the most acidic solution (pH = 2), respectively (Fig. S8a and b, ESI†), which is in good agreement with similar previously reported studies.^59,60^

**Fig. 6:**
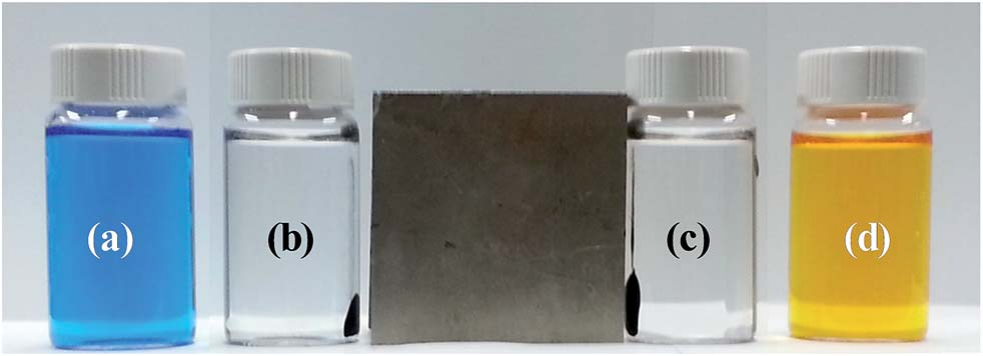
Photographs of aqueous solutions of (a) MB and (d) MO and the FeCo/GC NCs@MSNs-SH collected using a magnet in the aqueous solutions after the adsorption of the organic dyes, MB (b) and MO (c).

We also studied decoloration of water, which indicates efficient adsorption of dyes at low concentration. It is very important to completely remove pollutants at low concentration because this determines the concentration of residual pollutants in the water. We carried out an experiment with 20 mg of FeCo/GC NCs@MSNs-SH and 20 mL of each dye solution (10 ppm MB and 5 ppm MO). The FeCo/GC NCs@MSNs-SH were aged in the solutions for 5 min and the dye-adsorbed FeCo/GC NCs@MSNs-SH were collected using a magnet within 1 min so that the total contact time was about 6 min. Fig. 6 shows photographs of the MB (Fig. 6a) and MO (Fig. 6d) solutions and the samples after collection of the adsorbent by magnet (Fig. 6b for MB and 6c for MO). The polluted water became clear and colorless. All the adsorption rates were >99.5% which was the detection limit, representing complete removal of dyes. It is also worth mentioning that the FeCo/GC NCs@MSNs-SH was collected very quickly (within 1 min) and almost completely by magnet from the solutions due to the very high saturation magnetization of the FeCo/GC NCs (205 emu g^−1^).^43^ This result reveals the efficient adsorption and distinct removal of pollutants in aqueous solutions possible with the use of FeCo/GC NCs@MSNs-SH.

**Fig. 7:**
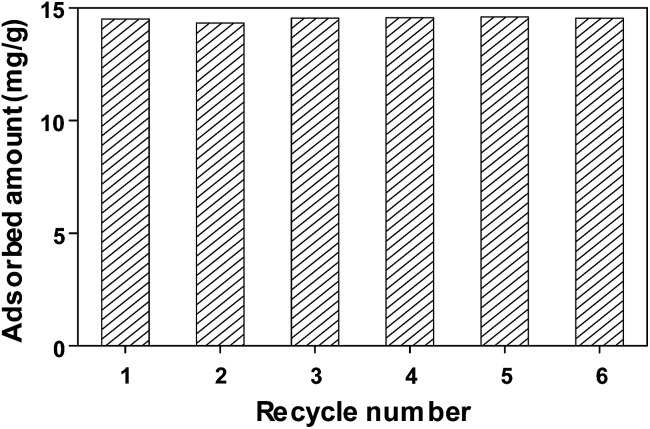
Regeneration study of FeCo/GC NCs@MSNs-SH for the adsorption of MO in water at room temperature.

We also investigated the feasibility of FeCo/GC NCs@MSNs-SH as a recyclable adsorbent by repeating adsorption and desorption of MO. The steps in each cycle were as follows. The FeCo/GC NCs@MSNs-SH was put into aqueous MO solution and stirred until equilibrium was reached at room temperature. Subsequently, the dye-adsorbed FeCo/GC NCs@MSNs-SH was separated from the suspension using a magnet, resulting in a clear solution. The loaded adsorbent was then transferred into methanol to release the adsorbed dye. Next, we again collected the dye-desorbed FeCo/GC NCs@MSNs-SH using the magnet. The result was almost clean, regenerated FeCo/GC NCs@MSNs-SH that was then used in the next adsorption experiment. As shown in Fig. 7, during the six adsorption/desorption cycles the adsorption capacity of the FeCo/GC NCs@MSNs-SH remained almost the same as the initial one (14.6 mg g^−1^). This demonstrated the recyclability of the FeCo/GC NCs@MSNs-SH. We also confirmed that the adsorbent was not grossly altered during the six recycling experiments based on TEM image of the FeCo/GC NCs@MSNs-SH after the experiments (Fig. S9, ESI†).

In another step, we additionally explored the functionality of the FeCo/GC NCs@MSNs-SH as an adsorbent of Hg^2+^ ions. The adsorption capacity was obtained by measuring the amount of residual Hg^2+^ ions in the solution after adsorption. Since the solution pH affects the adsorption of Hg^2+^ ions on the FeCo/GC NCs@MSNs-SH, we examined the influence of the pH with solutions of pH 2, 4, and 7 (basic solutions were excluded because Hg^2+^ ions form various species such as HgO at pH values greater than 7.^61^). The FeCo/GC NCs@MSNs-SH showed the highest adsorption at a solution of pH 4 (Fig. S8c, ESI†).^62^ Therefore, we selected the pH value of 4 for our subsequent Hg^2+^ adsorption experiments with the FeCo/GC NCs@MSNs and MSNs for comparison purposes. Table 1 shows that the adsorption capacity of FeCo/GC NCs@MSNs-SH was as high as 221.4 mg g^−1^ and was significantly higher than that of FeCo/GC NCs@MSNs (11.0 mg g^−1^) and of MSNs (2.3 mg g^−1^). A comparison between this work and other reported data from the literature (Table S4, ESI†) shows that the adsorption capacity of FeCo/GC NCs@MSNs-SH is among the highest values. The molar ratio of adsorbed Hg^2+^ ions to S atoms in FeCo/GC NCs@MSNs-SH was 1.25, exceeding the theoretical value of ‘1’. This means that one S atom interacts with more than one Hg^2+^ ion. The FeCo/GC NCs@MSNs-SH exhibited the greatest binding capacity for Hg^2+^ ions (among adsorbents with similar thiol content) ever reported.^50,51,63^ This can be attributed to the high accessibility of thiol groups to Hg^2+^ ions in the unique mesoporous structure and additional binding of Hg^2+^ ions at the available oxygen-donor sites on the silica surface.^64,65^ Thiol functionality provides unique capacity for Hg^2+^ ions while the superior magnetic response imparted by FeCo/GC NCs enables rapid separation of the adsorbed metal ions.

## 4. Conclusions

In summary, we successfully synthesized magnetic mesoporous silica nanospheres incorporated with FeCo/GC nanocrystals (FeCo/GC NCs@MSNs). The magnetic nanospheres showed very high stability in 35% HCl and NaOH (pH = 11) solutions, and could be separated from the matrix solutions within 1 min using a magnet. Surface functionalization of the FeCo/GC NCs@MSNs with thiol groups gave thiol-functionalized FeCo/GC NCs@MSNs (FeCo/GC NCs@MSNs-SH) that have sites efficient for binding Hg^2+^ ions. The FeCo/GC NCs@MSNs-SH were demonstrated to be multifunctional adsorbents for both organic dyes and Hg^2+^ ions, and a recyclable adsorbent for MO. Our magnetic nanocomposite system may offer a new form of highly stable and magnetically recyclable multifunctional adsorbents. These could provide effective, simultaneous separation of organic and inorganic pollutants from wastewater, thereby playing an important role in environmental remediation.

## Conflicts of interest

There are no conflicts to declare.

## Supplementary Material

RA-008-C7RA12240C-s001
