# Supplementary material for: Highly stable mesoporous silica nanospheres embedded with FeCo/graphitic shell nanocrystals as magnetically recyclable multifunctional adsorbents for wastewater treatment
Source: RSC Adv. 2018 Jan 3;8(2):1089–97. doi: 10.1039/c7ra12240c (PMC9077014; doi:10.1039/c7ra12240c)
Supplement: RA-008-C7RA12240C-s001 [file RA-008-C7RA12240C-s001.pdf]

Electronic Supplementary Material (ESI) for RSC Advances.
This journal is © The Royal Society of Chemistry 2017

Electronic Supplementary Information for

# Highly Stable Mesoporous Silica Nanospheres Embedded with FeCo/Graphitic Shell Nanocrystals as Magnetically Recyclable Multifunctional Adsorbents for Wastewater Treatment

Yonghoon Hong,‡[a] Da Jeong Kim,‡[a] In Ae Choi,[a] Mou Pal,[b] Gaehang Lee,*[c] Ki Min Nam,*[d] Won Seok Seo*[a]

[a]*Department of Chemistry, Sogang University, Seoul, 04107, Republic of Korea*

[b]*Instituto de Física, BUAP, Av. San Claudio y Blvd. 18 Sur Col. San Manuel, Ciudad Universitaria, C.P. 72570 Puebla, Mexico*

[c]*Korea Basic Science Institute and University of Science and Technology, Daejeon 34133, Republic of Korea*

[d]*Department of Chemistry, Mokpo National University, Jeonnam 58554, Republic of Korea*

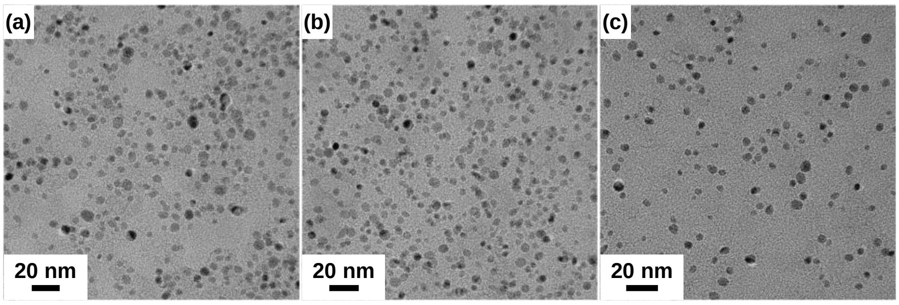


Fig. S1. TEM images of FeCo/GC NCs obtained from (a) 65 nm, (b) 130 nm, and (c) 270 nm FeCo/GC NCs@MSNs after HF treatment.

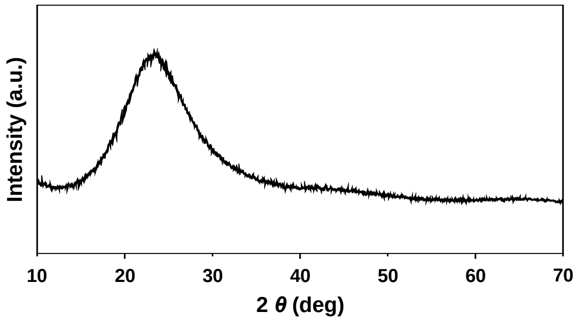


Fig. S2. XRD pattern of MSNs.

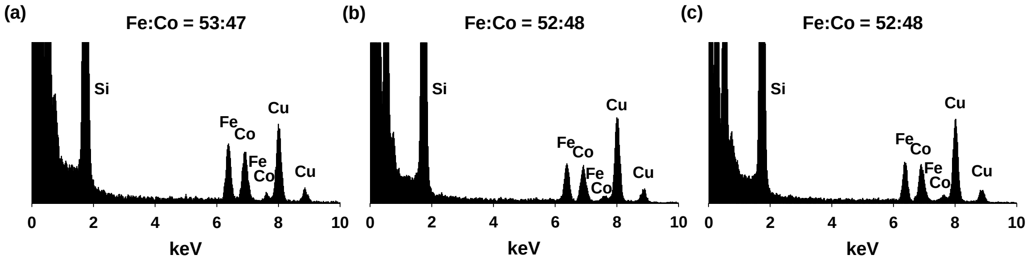


Fig. S3. EDX data of (a) 65 nm, (b) 130 nm, and (c) 270 nm FeCo/GC NCs@MSNs.

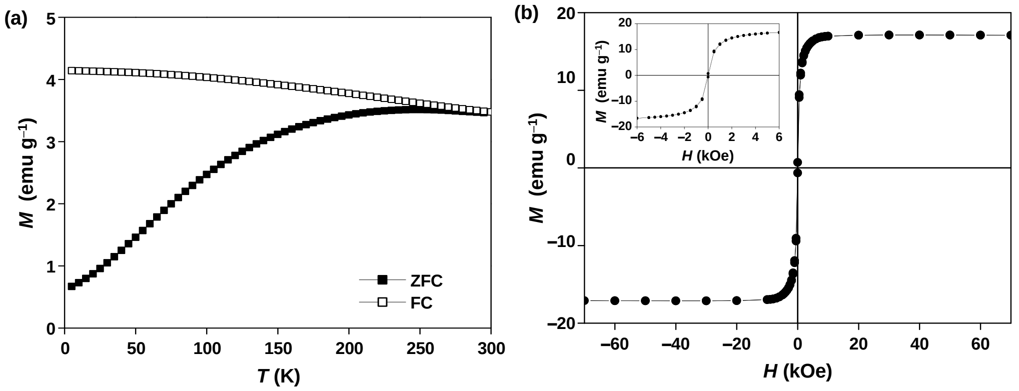


Fig. S4. (a) Temperature-dependent magnetization curves under an applied field of 100 Oe and (b) Field-dependent magnetization curves at 300 K for 130 nm FeCo/GC NCs@MSNs. Inset in (b) shows the loop on an enlarged x-axis scale.

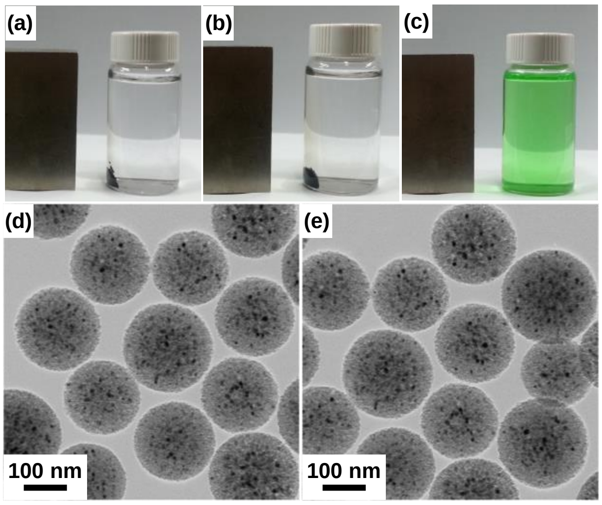


Fig. S5. Photographs of 130 nm (a, b) FeCo/GC NCs@MSNs-SH and (c) FeCo NCs@MSNs-SH in 35% HCl solutions (a, c) and a 1 mM NaOH (pH 11) solution (b). TEM images of FeCo/GC NCs@MSNs-SH stored over a monitoring period of a week in the (d) HCl and (e) NaOH solutions, respectively. FeCo/GC NCs@MSNs-SH exhibited stability against HCl or NaOH etching over a

monitoring period of a week. However, FeCo NCs@MSNs-SH having FeCo (being unencapsulated with a carbon shell) NCs turned the color to green in the HCl solution right after the addition due to the Fe and Co etching.

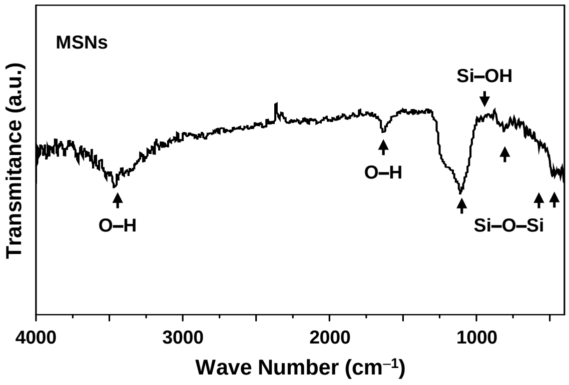


Fig. S6. FT-IR data of 130 nm MSNs.

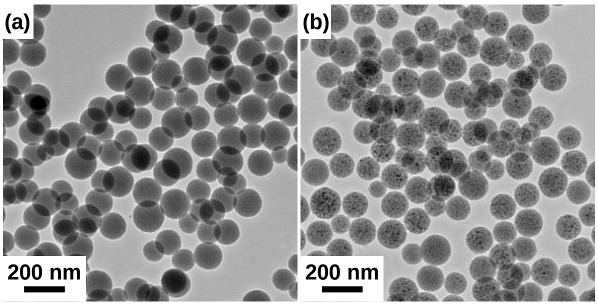


Fig. S7. TEM images of 130 nm (a) MSNs-SH and (b) FeCo/GC@MSNs-SH.

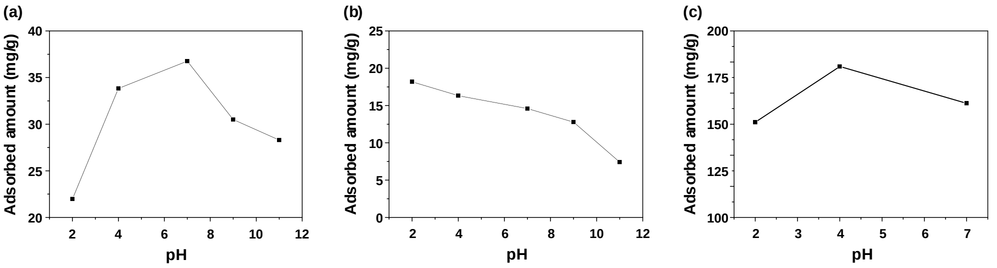


Fig. S8. Effect of pH on the adsorption of (a) MB, (b) MO, and (c) $Hg^{2+}$ onto the FeCo/GC NCs@MSNs-SH.

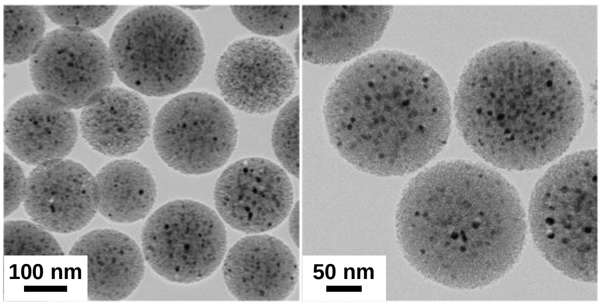


Fig. S9. TEM images of 130 nm FeCo/GC NCs@MSNs-SH after the six consecutive adsorption cycles for MO.

Table S1. Physicochemical properties of selected samples.

| Sample | BET surface area ($m^2/g$) | Pore volume ($cm^3/g$) | pore size (nm) |
|---|---|---|---|
| MSNs | 661 | 0.73 | 2.45 |
| FeCo/GC NCs@MSNs | 442 | 0.65 | 2.19 |

Table S2. Langmuir isotherm parameters for MB adsorption on various adsorbents.

| Sample | Langmuir model | | |
|---|---|---|---|
| | $q_{max}$ | $b$ | $R^2$ |
| MSNs | 20.33 | 1.54 | 0.99 |
| FeCo/GC NCs@MSNs | 28.99 | 2.97 | 0.99 |
| FeCo/GC NCs@MSNs-SH | 37.17 | 3.49 | 0.99 |

Table S3. Langmuir isotherm parameters for MO adsorption on various adsorbents.

| Sample | Langmuir model | | |
|---|---|---|---|
| | $q_{max}$ | $b$ | $R^2$ |
| MSNs | 3.33 | 0.38 | 0.97 |
| FeCo/GC NCs@MSNs | 13.39 | 0.48 | 0.98 |
| FeCo/GC NCs@MSNs-SH | 15.75 | 0.57 | 0.99 |

Table S4. Comparison of adsorption capacities of FeCo/GC NCs@MSNs-SH with different adsorbents.

| **Magnetic adsorbents for MB** | $q_e$ | *pH* | **References** |
|---|---|---|---|
| RGO–$MnFe_2O_4$ hybrid | 34.7 | – | S. Bai et al. (2012)[1] |
| Fe3O4@C | 52.5 | 7.0 | S. P. Wu et al. (2016)[2] |
| MMWCNT | 11.9 | 7.0 | J. L. Gong et al. (2009)[3] |
| M-MWCNTs | 45.8 | 7.0 | L. Ai et al. (2011)[4] |
| MGO | 275.9 | 9.0 | Y. F. Guo et al. (2016)[5] |
| CS/Mt-OREC | 9.7 | 7.0 | L. Zeng et al. (2015)[6] |
| ɤ-$Fe_2O_3$/C composites | 193.4 | – | J. Xiao et al. (2013)[7] |
| FeCo/GC NCs@MSNs-SH | 36.8 | 7.0 | This Study |
| **Magnetic adsorbents for MO** | $q_e$ | *pH* | **References** |
| m-CS/c-$Fe_2O_3$/MWCNTs | 61.4 | – | H. Y. Zhu et al. (2010)[8] |
| CS/Mt-OREC | 5.0 | 7.0 | L. Zeng et al. (2015)[6] |
| CANF | 102.0 | 4.0 | B. Tanhaei et al. (2015)[9] |
| ɤ-$Fe_2O_3$/chitosan | 28.5 | 2.9 | R. Jiang et al. (2012)[10] |
| AC/$NiFe_2O_4$ | 93.5 | 3.0 | T. Jiang et al. (2015)[11] |
| FeCo/GC NCs@MSNs-SH | 14.6 | 7.0 | This study |
| **Magnetic adsorbents for Hg(II)** | $q_e$ | *pH* | **References** |
| MAF-SCMNPs | 240.0 | 6.0 | S. Bao et al. (2017)[12] |
| $Fe_3O_4$@$Cu_3(btc)_2$ | 158.2 | 6.0 | F. Ke et al. (2017)[13] |
| $Fe_3O_4$@$SiO_2$–SH | 148.8 | 6.5 | S. Zhang et al. (2013)[14] |
| PR-MNPs | 133 | 4.0 | J. Song et al. (2011)[15] |
| CG-MCS | 220.1 | 7.0 | Y. Wang et al. (2013)[16] |
| AEPE-PS-MPs | 28.7 | 7.0 | K. Jainae et al. (2015)[17] |
| TETA-PGMA | 468 | 6.0 | Y. Wang et al. (2016)[18] |
| Thiol-functionalized MGO | 30.9 | – | J. Bao et al. (2013)[19] |
| HMSMCs | 62.8 | 6.5 | X. Zhang et al. (2015)[20] |
| MGO | 59.9 | 6.0 | Y. F. Guo et al. (2016)[5] |
| rGO-Fe(0)-$Fe_3O_4$ | 22.0 | 7.0 | P. Bhunia et al. (2012)[21] |
| FeCo/GC NCs@MSNs-SH | 221.4 | 4.0 | This study |

## References


1 S. Bai, X. Shen, X. Zhong, Y. Liu, G. Zhu, X. Xu and K. Chen, *Carbon*, 2012, **50**, 2337–2346.

2 S. P. Wu, J. C. Huang, C. H. Zhuo, F. Y. Zhang, W. C. Sheng and M. Y. Zhu, *J. Inorg. Organomet. Polym. Mater.*, 2016, **26**, 632–639.

3 J. L. Gong, B. Wang, G. M. Zeng, C. P. Yang, C. G. Niu, Q. Y. Niu, W. J. Zhou and Y. Liang, *J. Hazard. Mater.*, 2009, **164**, 1517–1522.

4 L. Ai, C. Zhang, F. Liao, Y. Wang, M. Li, L. Meng and J. Jiang, *J. Hazard. Mater.*, 2011, **198**, 282–290.

5 Y. F. Guo, J. Deng, J. Y. Zhu, X. J. Zhou and R. B. Bai, *RSC Adv.*, 2016, **6**, 82523–82536.

6 L. Zeng, M. Xie, Q. Zhang, Y. Kang, X. Guo and H. Xiao, *Carbohydr. Polym.*, 2015, **123**, 89–98.

7 J. Xiao, L. Qiu, X. Jiang, Y. Zhu, S. Ye and X. Jiang, *Carbon*, 2013, **59**, 372–382.

8 H. Y. Zhu, R. Jiang, L. Xiao and G. M. Zeng, *Bioresour. Technol.*, 2010, **101**, 5063–5069.

9 B. Tanhaei, A. Ayati, M. Lahtinen and M. Sillanpaa, *Chem. Eng. J.*, 2015, **259**, 1–10.

10 R. Jiang, Y.-Q. Fu, H.-Y. Zhu, J. Yao and L. Xiao, *J. Appl. Polym. Sci.*, 2012, **125**, E540–E549.

11 T. Jiang, Y. D. Liang, Y. J. He and Q. Wang, *J. Environ. Chem. Eng.*, 2015, **3**, 1740–1751.

12 S. Bao, K. Li, P. Ning, J. Peng, X. Jin and L. Tang, *Appl. Surf. Sci.*, 2017, **393**, 457–466.

13 F. Ke, J. Jiang, Y. Li, J. Liang, X. Wan and S. Ko, *Appl. Surf. Sci.*, 2017, **413**, 266–274.

14 S. Zhang, Y. Zhang, J. Liu, Q. Xu, H. Xiao, X. Wang and J. Zhou, *Chem. Eng. J.*, 2013, **226**, 30–38.

15 J. Song, H. Kong and J. Jang, *J. Colloid Interface Sci.*, 2011, **359**, 505–511.

16 Y. Wang, Y. Qi, Y. Li, J. Wu, X. Ma, C. Yu and L. Ji, *J. Hazard. Mater.*, 2013, **260**, 9–15.

17 K. Jainae, N. Sukpirom, S. Fuangswasdi and F. Unob, *J. Ind. Eng. Chem.*, 2015, **23**, 273–278.

18 Y. Wang, Y. Zhang, C. Hou, X. He and M. Liu, *J. Taiwan Inst. Chem. E.*, 2016, **58**, 283–289.

19 J. Bao, Y. Fu and Z. H. Bao, *Nanoscale Res. Lett.*, 2013, **8**, 486–492.

20 X. Zhang, T. Wu, Y. Zhang, D. H. L. Ng, H. Zhao and G. Wang, *RSC adv.*, 2015, **5**, 51446–51453.

21 P. Bhunia, G. Kim, C. Baik and H. Lee, *Chem. Commun.*, 2012, **48**, 9888–9890.
